# From Cisplatin-Containing Sequential Radiochemotherapy towards Concurrent Treatment for Patients with Inoperable Locoregional Non-Small Cell Lung Cancer: Still Unanswered Questions

**DOI:** 10.1155/2010/506047

**Published:** 2010-12-28

**Authors:** C. C. E. Koning, J. S. A. Belderbos, A. L. J. Uitterhoeve

**Affiliations:** ^1^Department of Radiation Oncology, Academic Medical Center (AMC), University of Amsterdam, P.O. Box 22660, 1100 DD Amsterdam, The Netherlands; ^2^Department of Radiation Oncology, Netherlands Cancer Institute/Antoni Van Leeuwenhoek Hospital, P.O. Box 90203, 1006 BE Amsterdam, The Netherlands

## Abstract

Radiotherapy has been the mainstay of the treatment of stage III non-small cell lung cancer (NSCLC) patients. In the early nineties, combined treatment with chemotherapy was introduced. In 1995, a meta-analysis showed improved treatment outcome of the sequential use of radiochemotherapy (RCT) compared to radiotherapy alone, provided cisplatin was part of the chemotherapy course. Concurrent RCT compared to radiotherapy only yielded the same improvements of 4% in the 2-year and 2% in the 5-year overall survival rates. Just recently, two meta-analyses demonstrated that concurrent RCT is definitely superior to sequential RCT in terms of local control and 2-, 3-, and 5-year survival. However, several unanswered questions remain concerning the optimal chemotherapy regimen and radiotherapy doses and techniques in terms of treatment outcome and toxicity profile. Arguments supporting a daily low-dose cisplatin scheme are presented because of comparable radiosensitizing characteristics and favourable side effects. Increasing radiotherapy doses applied according to up-to-date techniques and combinations with new biologicals might lead to further treatment improvements.

## 1. Introduction

Until the nineties radiotherapy alone was the standard treatment for stages IIIA and IIIB non-small cell lung cancer (NSCLC). With the standard dose of 60 Gy in 30 fractions, results in terms of 1-, 2-, and 5-year survival rates were poor [1]. For NSCLC a dose-effect relationship exists: the higher the radiation dose, the greater the probability of tumour control [2]. Kong et al. reported improved local control and survival for patients irradiated with doses above 74 Gy in the dose escalation trial of the University of Michigan. Strategies to improve the treatment results include increasing doses of radiotherapy and decreasing overall treatment time [3]. A different option is to combine radiotherapy with chemotherapy. The first report on improved 1- and 2-year survival after adding chemotherapy to the irradiation was published by Dillman et al. in 1990 [4].

## 2. Sequential Radiochemotherapy

The strategy of radiotherapy only changed essentially after the publication of the meta-analysis by the Non-small Cell Lung Cancer Collaborative Group in 1995 [5].

Radiotherapy preceded by (usually) two courses of chemotherapy yielded an improvement of the 2-year overall survival rate from 21% to 25%. The 5-year survival increased from 6% to 8% provided that the chemotherapy regimen included cis-diamminedichloroplatinum II (cisplatin). Without cisplatin no improvements in treatment outcome were achieved.

The effect was explained by a reduction of distant metastases. Until now this effect of a lower distant metastasis rate was observed in one study only [6]. In this study, Le Chevalier et al. compared radiotherapy alone to chemotherapy and radiotherapy. However, patients with adenocarcinoma were excluded. Since an important proportion of the NSCLC patients were not included, the results might not be representative. The 3-year survival rate was 12% for the combination arm versus 4% for the radiotherapy arm (*P* < .02). To our knowledge, these results have never been confirmed. 

Until recently sequential cisplatin-containing radio-chemotherapy has been the standard treatment for inoperable stage IIIA and B disease. Various chemotherapy schedules have been applied, but the treatment outcome did not differ significantly.

## 3. Concurrent Radiochemotherapy

A different approach of combining chemotherapy and radiotherapy was studied in Europe. After phase I and phase II studies, the EORTC started a 3-arm phase III trial comparing split-course radiotherapy of 55 Gy using the same radiotherapy scheme, concurrently combined with 30 mg/m^2^ cisplatin once a week or 6 mg/m^2^ daily in 1984 [7–9].

The results were published in 1992 [10]. The most important conclusions were as follows: weekly cisplatin administration did not yield a statistically significant improvement; 6 mg/m^2^ cisplatin daily added to radiotherapy improved survival; this gain was related to improved local progression-free survival. There was no effect on the distant metastasis rate, and late toxicity was not increased. These data demonstrated that cisplatin improved the radiotherapy effect by radiosensitization. The most frequently reported acute side effects were nausea and vomiting. In 1992, Trovo et al. also published their randomised phase III study [11]. Three weeks of radiotherapy, to a dose 45 Gy, were compared to the same radiotherapy dose with the addition of 6 mg/m^2^ cisplatin daily. No positive effect on survival was found, however, maybe due to the low radiation dose prescribed.

After the introduction of the newly developed antiemetics, the 5-HT3 antagonists, several other groups reported their results with concurrent radiochemotherapy in NSCLC. Repeatedly two full-dose chemotherapy courses in a variety of cisplatin, doublets or triplets were combined with radiotherapy. 

All phase III trials were included in a meta-analysis by Aupérin et al, indicating a 4% survival gain at 2 years and 2% at 5 years, for concurrent chemoradiation versus radiotherapy alone, a comparable improvement as observed with the sequential combination [12]. A Cochrane meta-analysis confirmed these conclusions [13]. The concomitantly applied chemotherapy is considered to have radiosensitizing capacities.

In the meantime EORTC study 08844 was followed by phase I-II studies in which the radiotherapy was intensified to reach an accelerated fractionation scheme (66 Gy in 24 fractions) in a reduced overall treatment time (32 days) [14, 15]. This radiobiologically equivalent dose of at least 78.5 Gy/2 Gy (BED *α*/*β* = 2) with daily low-dose cisplatin before each fraction during the entire course was feasible and safe [16].

## 4. Sequential versus Concurrent Radiochemotherapy

Several groups addressed the question of which type of radiochemotherapy to prefer: the sequential or the concurrent combination. In several trials improved 1- and 2-year overall survival rates in favour of the concurrent arm were reported [17–23].

Most of these trials were included in a new meta-analysis based on individual patient data by Aupérin et al, who concluded that concurrent radiochemotherapy yielded superior results compared to the sequential combinations: 2-, 3-, and 5-year survival rates of 35.6%, 23.8%, and 15.1% versus 30.3%, 18.1%, and 10.6%, respectively (*P* = .004) [24]. This improved survival was accomplished because of an improved locoregional control. There were no significant differences between the regimens: single or double high-dose chemotherapy or daily low-dose cisplatin. No differences in distant metastasis rate were observed between the two approaches. Within a few months a meta-analysis was published by O'Rourke et al. reporting a 10% absolute survival benefit at two years [25]. The most important acute but manageable side effect was esophagitis grade 3 to 4 in 18% of the patients treated with concurrent radiochemotherapy versus 4% in the patients treated with sequential RCT.

The conclusion was finalized: concurrent radio-chemotherapy became the new standard treatment for locally advanced nonmetastasized stages of NSCLC. It should be realized that the trial data were collected in a period before routine staging with FDG-PET and MRI of the brain. In the future improved selection of the patients will contribute to the treatment results.

## 5. Unanswered Questions

New questions arise, however: what is the optimal combination of chemotherapy used concurrently with high-dose radiotherapy? To answer this question we took a closer look at the toxicities of the trials included in the meta-analysis of Aupérin et al. [24]. Arbitrarily we selected those studies that included at least 50 patients in the concurrent arm to assemble more reliable data. In the Furuse trial 80 mg/m^2^ cisplatin was given on days 1 and 29, combined with vindesine 3 mg/m^2^ on days 1, 8, 29, and 36 and mitomycin 8 mg/m^2^ on days 1 and 29 with 56 Gy, 2 Gy/fraction, split-course radiotherapy [20]. In the French trial cisplatin was prescribed during weeks 1 and 5 using a daily dose of 20 mg/m^2^ together with 50 mg/m^2^ etoposide. The radiotherapy dose was 66 Gy in 33 fractions in 6.5 weeks, followed by two consolidation cycles consisting of cisplatin and vinorelbine [19]. The EORTC trial prescribed 6 mg/m^2^ cisplatin daily combined with 2.75 Gy radiotherapy, total dose 66 Gy/24 fractions, 5 fractions a week [23].

The study of Zatloukal et al., missing in the meta-analysis, prescribed 80 mg/m^2^ cisplatin on day 1 and vinorelbine 25 mg/m^2^ on days 1, 8, and 15, repeated every 28 days. The radiotherapy, 60 Gy/30 fractions during 6 weeks, was started on day 4 resulting in a weekend rest between the chemotherapy administration and start of radiotherapy [22]. The treatment results in terms of 2- and 3-year overall survival are plotted in Table 1 and seem comparable to the daily low-dose cisplatin outcome. For the RTOG study no full paper has been published, and important data on toxicity are lacking [18].

The treatment-related toxicities are listed in Table 1 as well. In general high-dose cisplatin was accompanied by more frequent haematological toxicity. Esophagitis was 3% in the split-course radiotherapy scheme and more or less equal (18%) for the other varieties with exception for the study by Fournel et al. Nausea and vomiting was much lower for the daily low-dose cisplatin combination. Other toxicities such as neuropathy, renal toxicity, and pneumonitis were reported in different ways and were generally infrequent if presented. Fournel reported a striking toxic death rate of 11%, maybe also related to the combination with consolidation chemotherapy treatment. Many patients were not capable to receive the two additional courses after finishing the concurrent radiochemotherapy.

The mild toxicity profile of daily 6 mg/m^2^ cisplatin and competitive clinical outcome with accelerated radiotherapy compared to full-dose concurrent RCT supports the use of this scheme, although randomized prospective clinical trials will finally yield the proof. To our knowledge until now no such studies have been performed.

In the study of Belderbos et al. the radiation dose was the highest compared to the schemes used in the other reports [23].

Trials studying the role of induction or consolidation chemotherapy show disappointing results [26, 27].

## 6. Other Arguments in Favour of Daily Low-Dose Cisplatin 

Since the incidence of NSCLC is high among elderly patients (at least 40% of the patients are older than 70 years in The Netherlands) and many of them have a smoking history, the majority has severe comorbidities. Epidemiological studies show that, with increasing age, the percentage of people treated with chemotherapy decreases. However, age is not an independent prognostic factor in stage III and IV NSCLC [28–30]. Elderly patients with marginal renal function (creatinine clearance <70 mL/min) or marginal cardiac function (hyperhydration contra-indicated) are eligible for administration of daily low-dose cisplatin, while administration of full-dose chemotherapy is often contraindicated. Combination of concurrent daily cisplatin with radiation appears to be a good alternative, especially in these elderly, frail patients [31, 32]. 

For patients with head and neck tumours, hearing loss due to cisplatin administration was studied and reported low in case of daily low-dose cisplatin compared to high-dose courses [33]. Preclinical studies on RCT support the use of daily administration for optimal radiosensitizing effects [34]. However, when logistics make this daily administration too complicated, a weekly cisplatin dose can be considered (40 mg/m^2^) to conform with the guidelines for radiochemotherapy in patients with cervical cancer [35].

Alternative platinum compounds such as carboplatin have not yielded the same positive results as cisplatin [36, 37]. 

To our opinion the favourable toxicity profile makes the daily low-dose cisplatin with high-dose accelerated radiotherapy combination suitable for addition of new antitumour agents. Many new biologicals have entered the therapeutic domain, several were combined with concurrent RCT regimens. Some combinations appear to be too toxic like the vascular epithelial growth factor antibody bevacizumab [38] or ineffective as the tyrosine kinase inhibitor (TKI) gefitinib [39]. The feasibility of adding the epidermal growth factor receptor inhibitor cetuximab has been recently reported for NSCLC patients [40]. Results of a running randomized phase II trial comparing daily cisplatin with or without cetuximab need to be awaited. Studies on the antifolate pemetrexed are under way [41]. Until now no definite data can be reported.

Other topics for future research are RCT with more sophisticated radiotherapy techniques allowing possibly higher tumour doses and/or lower toxicities in surrounding healthy tissues. For patients with larger tumor volumes, the possibilities to increase the radiation dose were limited by normal tissue constraints (esophagus and spinal cord). Intensity-modulated radiotherapy (IMRT) has the potential benefit to further increase the dose that can be safely prescribed in lung cancer patients due to a better conformity index and a steeper dose falloff [42–44]. This technique has been widely introduced recently.

## 7. Conclusions

In conclusion after two decades of mainly sequentially combined treatment, concurrent radiochemotherapy is nowadays the standard treatment. Since the daily low-dose cisplatin can be safely combined with high-dose accelerated radiotherapy, this treatment option is still very appealing. In general oncologists should offer the least harmful treatment to their patients in case several treatment options are available with equal efficacy.

This approach, delivered in a short overall treatment time, is suitable for the elderly and for patients with comorbidities as well. It also offers the opportunity to combine this concomitant radiochemotherapy with new agents.

## Figures and Tables

**Table 1:** Survival data and toxicities of concurrent treatment regimens with >50 patients included.

Author of publication	Zatloukal et al. [22]	Fournel et al. [19]	Furuse et al. [20]	Belderbos et al. [23]
*Number of patients*	102	205	314	158
*Survival data (%)*				
Overall survival 2 years	34.2	26.5	34.6	34
Overall survival 3 years	18.6	18.6	22.3	29.2
*Acute toxicity (%)*				
Neutropenia grade 3 + 4	65	77	100	2
Thrombocytopenia	6	16	53	0
Anaemia	12	20	10	0
Esophagitis	18	32	3	17
Nausea/vomiting	39	24	22	6
